# Mineral Element Insiders and Outliers Play Crucial Roles in Biological Evolution

**DOI:** 10.3390/life12070951

**Published:** 2022-06-24

**Authors:** Eli K. Moore, Daniella L. Martinez, Naman Srivastava, Shaunna M. Morrison, Stephanie J. Spielman

**Affiliations:** 1 Department of Environmental Science, School of Earth and the Environment, Rowan University, Glassboro, NJ 08028, USA; daniellalynn425@gmail.com; 2 Department of Biological Sciences, College of Science and Mathematics, Rowan University, Glassboro, NJ 08028, USA; namansri468@gmail.com (N.S.); stephanie.spielman@gmail.com (S.J.S.); 3 Earth and Planets Laboratory, Carnegie Institution for Science, Washington, DC 20015, USA; smorrison@carnegiescience.edu; 4 Childhood Cancer Data Lab, Alex’s Lemonade Stand Foundation, Bala Cynwyd, PA 19004, USA

**Keywords:** mineral elements, network analysis, localities, Earth crust, metal cofactors

## Abstract

The geosphere of primitive Earth was the source of life’s essential building blocks, and the geochemical interactions among chemical elements can inform the origins of biological roles of each element. Minerals provide a record of the fundamental properties that each chemical element contributes to crustal composition, evolution, and subsequent biological utilization. In this study, we investigate correlations between the mineral species and bulk crustal composition of each chemical element. There are statistically significant correlations between the number of elements that each element forms minerals with (#-mineral-elements) and the log of the number of mineral species that each element occurs in, and between #-mineral-elements and the log of the number of mineral localities of that element. There is a lesser correlation between the log of the crustal percentage of each element and #-mineral-elements. In the crustal percentage vs. #-mineral-elements plot, positive outliers have either important biological roles (S, Cu) or toxic biological impacts (Pb, As), while negative outliers have no biological importance (Sc, Ga, Br, Yb). In particular, S is an important bridge element between organic (e.g., amino acids) and inorganic (metal cofactors) biological components. While C and N rarely form minerals together, the two elements commonly form minerals with H, which coincides with the role of H as an electron donor/carrier in biological nitrogen and carbon fixation. Both abundant crustal percentage vs. #-mineral-elements insiders (elements that follow the correlation) and less abundant outsiders (positive outliers from the correlation) have important biological functions as essential structural elements and catalytic cofactors.

## 1. Introduction

Earth’s crust is composed of a wide range of abundant rock-forming elements and trace elements [1,2]. The unique properties of each crustal element results in differential partitioning and diverse chemical relationships among the planet’s fundamental building blocks [3]. The abundance, availability, and atomic properties of chemical elements resulted in their “natural selection” for specific biochemical roles and functions [4]. Light metals that act as electrolytes (Na, Mg, K, Ca) and non-metals that can form stable organic compounds, particularly the big six biological elements (C, H, N, O, P, S), have higher availability in seawater than trace elements which can be used as catalysts (e.g., Fe, Mn, Cu, Ni, Co, Zn, V, Mo [4,5]). There is mounting evidence that Earth’s inorganic components, particularly transition metals, were crucial drivers in the abiotic synthesis of metabolic precursors and electron transfer processes from which life originated and evolved [6,7,8]. Indeed, the paleomineralogy of the Hadean eon (>4.0 billion years ago; Ga) included many common minerals that incorporated trace elements as minor constituents, resulting in great diversity of reactive sites on mineral surfaces to promote prebiotic chemical interactions [9].

Available chemical elements in aqueous solutions largely originate from the lithosphere due to physical and chemical continental weathering processes over geologic time [10,11]. Similar to the impact of the abundance of chemical elements on biological utilization, the abundance of chemical elements in Earth’s crust influences the diversity of different minerals that each element occurs in [12]. Furthermore, planetary redox conditions that have impacted biological evolution throughout Earth history [13,14,15] are also recorded in the mineral record, including biologically mediated minerals [16,17,18,19]. Minerals show different chemical interactions between various elements, and these interactions can be recurring in Earth’s crust [20,21]. The distribution of each element across Earth’s surface mineralogy and specific element interactions within mineral structures can be used to understand the intrinsic properties each element contributes to crustal composition, evolution, and subsequent biological utilization as has been performed recently for cobalt, vanadium, and cadmium [22,23,24].

The estimated bulk composition of Earth’s crust is primarily made up of abundant silicates, aluminum oxides, iron oxides, calcium oxides, and other trace elements [2,25]. While various abundant crustal elements have important biological functions, it is not clear if crustal abundance is the main driver of each element’s mineral distribution in terms of the number of mineral species of which the element is an essential constituent and the number of localities that contain said minerals, and if crustal abundance and mineral diversity of chemical elements correlate with biological utilization. The essential biological elements carbon (C), hydrogen (H), and nitrogen (N) are not included in the Rudnick and Gao (2003) recommended composition of the bulk continental crust. However, C, H, and N have greater mineral diversity than many crustal trace elements [26,27,28]. In this study, we compiled the number of mineral species, mineral localities, and chemistry data for each mineral-forming element from the Mineral Evolution Database (MED; https://rruff.info/evolution/; accessed on 3 February 2020 [29]). Statistical analyses were then performed on these data to identify potential trends between mineral element distribution, element crustal abundance, and biological function. Mineral chemistry network analysis was also performed to compare the evolution of various low abundance outlier elements with C, H, and N.

## 2. Materials and Methods

We used the R package dragon to construct mineral networks analyze mineral species, locality, and chemistry data from the MED ([19,29]; https://rruff.info/evolution/; accessed on 3 February 2020). Mineral species and their ideal chemical formulas are as defined by the International Mineralogical Association (IMA; https://rruff.info/ima/; accessed on 3 February 2020; [30]). We calculated the numbers of elements that each element forms minerals with (#-mineral-elements), defined as all the elements that occur in the IMA-approved chemical formula of a mineral species of a particular element, excluding the element itself [31,32,33]. For example, the carbon-containing mineral abellaite [NaPb_2_(CO_3_)_2_(OH)] contains five different elements (Na, Pb, C, O, H). The carbon-containing mineral siderite (FeCO_3_) contains three chemical elements (Fe, C, O). If you add up all the elements of those two minerals (Na, Pb, C, O, H, Fe), you have five elements total, excluding carbon. In total, there are 50 chemical elements in various combinations in the 417 total carbon minerals in this analysis with unambiguous chemical formulas.

Linear regressions were performed on mineral species, locality, chemistry, and elemental abundance data using R, and associated data wrangling and visualization were performed using the R tidyverse suite of data science packages [34]. Adherence to linear regression assumptions were performed using the R package performance [35], and log axis transformations were applied when necessary to meet model assumptions. For all linear regressions, we excluded short-lived radioactive elements, such as Tc, Po, At, Ra, and Pu, from all analyses, because they were not present in sufficient quantities to form minerals after the early Hadean Eon (>4.3 Ga). We additionally excluded any elements that form fewer than five minerals.

The crustal weight percent abundance of each element for the bulk continental crust was compiled from Rudnick and Gao (2003). Mineral chemistry networks were constructed using the R package dragon [19]. The networks included low-abundance outliers (scandium, Sc; gallium, Ga; bromine, Br; ytterbium, Yb) of the linear correlations between the crustal weight percent abundance and #-mineral-elements. The low-abundance outlier elements were used as the focal elements for additional mineral chemistry networks. Mineral chemistry networks were also constructed using essential biological elements C, H, and N that are omitted from the Rudnick and Gao (2003) crustal composition as focal elements.

The mineral chemistry networks included two types of nodes: minerals (represented by colored circles), and the constituent elements of each mineral (represented by colored circles with the chemical symbol of the element). In the network, each mineral has a network connection (referred to as an “edge”) with each of the elements in the mineral’s chemical formula. For example, the mineral chalcopyrite (CuFeS_2_) has network edges connected to Cu, Fe and S. Louvain community detection cluster analysis [36] was performed using dragon to identify associations among minerals and elements in the C, H, and N combined mineral chemistry network.

### Code and Data Availability

All code and associated data needed to reproduce analyses are available from and documented in the repository https://github.com/spielmanlab/element_network_analysis; accessed on 3 February 2020. Crustal weight percent abundances calculated from Rudnick and Gao (2003) are available, along with associated code, in the R dragon package version >1.2.0 (https://github.com/sjspielman/dragon; accessed on 3 February 2020).

## 3. Results

**Figure 1:**
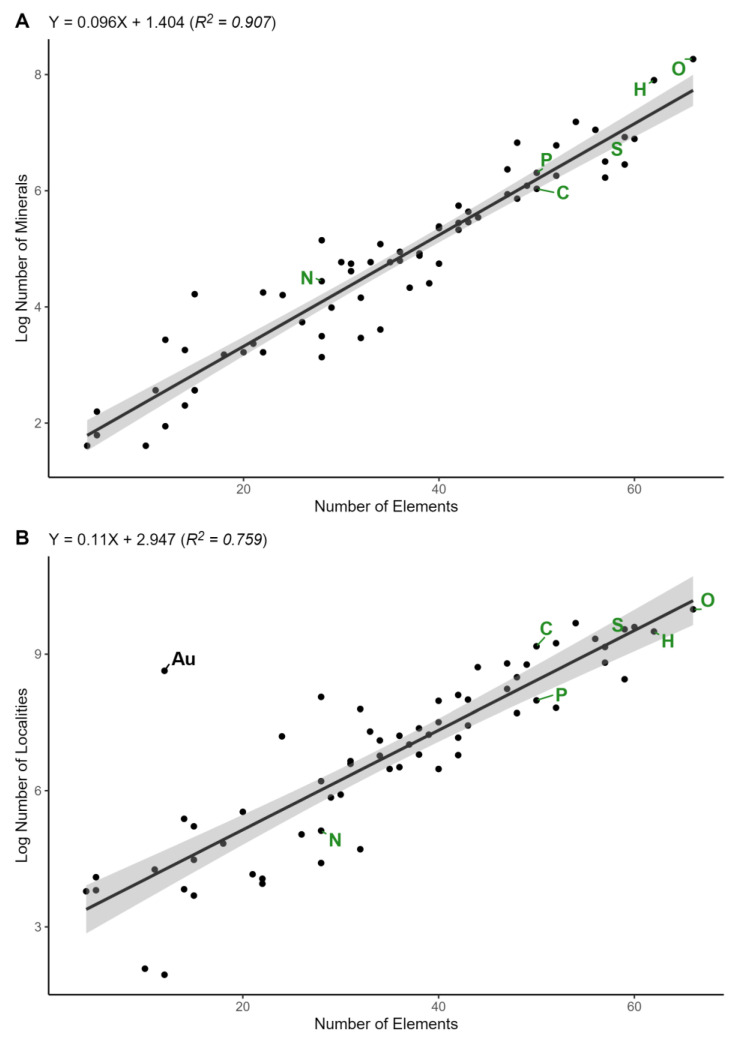
(**A**) Plot of the number of elements that each chemical element forms minerals with (#-mineral-elements) vs. the log of the number of minerals that each element occurs in the idealized chemical formula. (**B**) Plot of #-mineral-elements vs. the log of the number of mineral localities that each element occurs at. Essential biological elements (carbon, C; hydrogen, H; nitrogen, N; oxygen, O; phosphorus, P; sulfur, S) are labeled in green font and the abundant positive outlier non-biological element (gold, Au) is labeled in black font. A mineral locality is a location where a mineral occurs.

We observed a strong positive relationship (R^2^ = 0.907; *p* = 9.82 × 10^−35^) between #-mineral-elements and the log of the number of mineral species that each element occurs in (Figure 1A). Oxygen (O) forms minerals with 66 other elements and occurs in 3892 mineral species in this analysis (MED; https://rruff.info/evolution/; accessed on 3 February 2020; [29]), which is the largest number of elements that any element forms minerals with and the largest number of mineral species that any element occurs in [31,32,33]. Conversely, rare earth elements (REEs) occur in very few mineral species idealized chemical formulas and form minerals with a limited number of other elements, despite the fact that many different mineral natural kind clusters contain REEs based on their non-ideal atomic structures [37].

Similarly, we observed a strong positive relationship (R^2^ = 0.759; *p* = 1.81 × 10^−21^) between #-mineral-elements and the log of the number of localities that each element occurs at in each of its mineral species (Figure 1B). The hard base elements O and fluorine (F), and intermediate base elements sulfur (S) and chlorine (Cl) are among the elements that form minerals with the most other elements and occur at the largest number of localities. Hard acids and intermediate acids include elements that form minerals with many other elements and occur at many localities, and hard/intermediate acids also include elements that form minerals with few other elements and occur at a few localities (Table A1). In general, soft acids and soft bases form minerals with fewer other elements and occur at fewer localities than their intermediate and hard counterparts.

**Figure 2:**
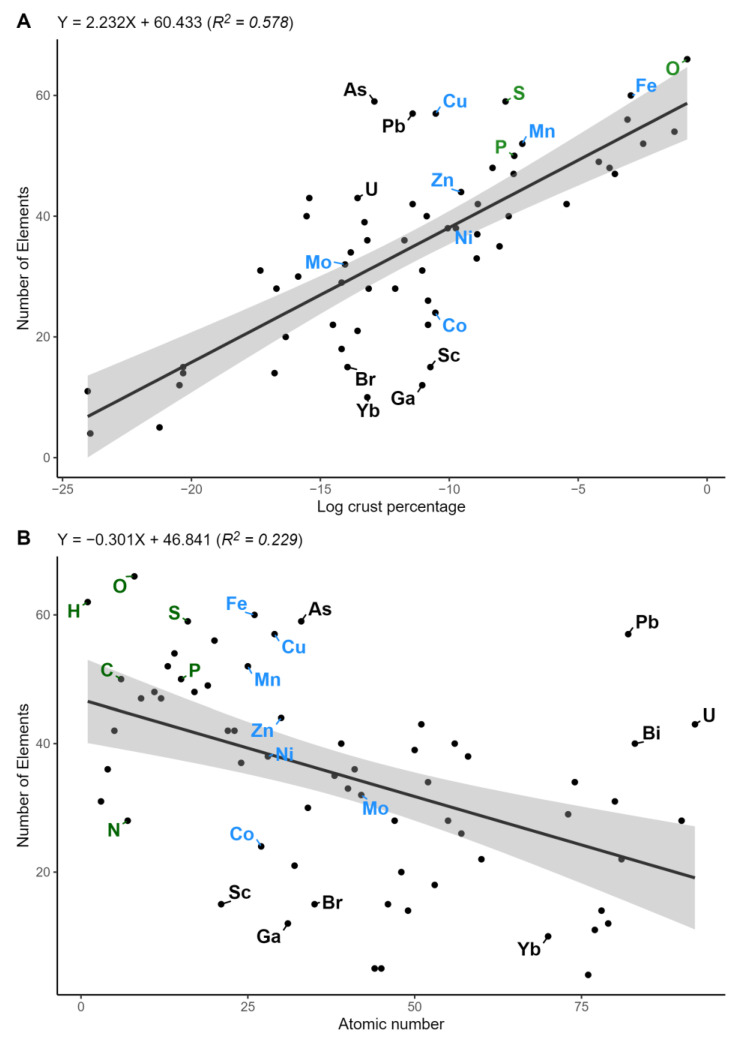
(**A**) Plot of the log of crustal percentage of each element (crustal abundance data compiled from Rudnick and Gao, 2003) vs. number of elements (#-mineral-elements). (**B**) Plot of the atomic number of each element vs. #-mineral-elements. Essential biological elements (carbon, C; hydrogen, H; nitrogen, N; oxygen, O; phosphorus, P; sulfur, S) are labeled in green font (the following essential biological elements were not included in the Rudnick and Gao (2003) study and subsequently not included in (**A**): carbon, C; hydrogen, H; nitrogen, N). Non-biological positive and negative outlier elements (arsenic, As; lead, Pb; Scandium, Sc; Gallium, Ga; ytterbium, Yb; bromine, Br; bismuth, Bi; uranium, U) are labeled in black font. Biological metals (iron, Fe; manganese, Mn; copper, Cu; zinc, Zn; nickel, Ni; molybdenum, Mo; cobalt, Co) are labeled in blue font.

There is a moderately positive relationship (R^2^ = 0.578; *p* = 1.8 × 10^−12^) between the log of the crustal weight percent abundance of each element and #-mineral-elements (Figure 2A). However, the R^2^ value of the crustal abundance and number of mineral-forming elements correlation (0.578) is lower than those shown in Figure 1, and there are various outlier elements from the overall trend. The elements S, copper (Cu), lead (Pb), and arsenic (As) all form minerals with a larger number of other elements than expected in the trend, while the elements Sc, Ga, Br, and Yb (and other REEs) all form minerals with fewer elements than expected in the trend (note: despite occurring in few minerals, gallium typically occurs as a minor element in numerous aluminum minerals [38]). For example, arsenic (As) has a crustal weight percent abundance of 2.49 × 10^−4^% and forms minerals with 59 other elements, while Sc has a crustal weight abundance of 2.18 × 10^−3^% and forms minerals with 15 other elements. There is a negative correlation with a lower R^2^ value than the other correlations described above (R^2^ = 0.229; *p* = 5.5 × 10^−5^) between element atomic number and #-mineral-elements (Figure 2B). The essential biological elements C, H, O, P, and S follow the general trend that lower atomic number elements form minerals with a larger number of elements than higher-atomic-weight elements, but N forms minerals with fewer elements than do all other elements with an atomic number <21 (Sc). Lead is the highest-atomic-weight outlier, which forms minerals with many more elements than other high-atomic-weight elements.

**Figure 3:**
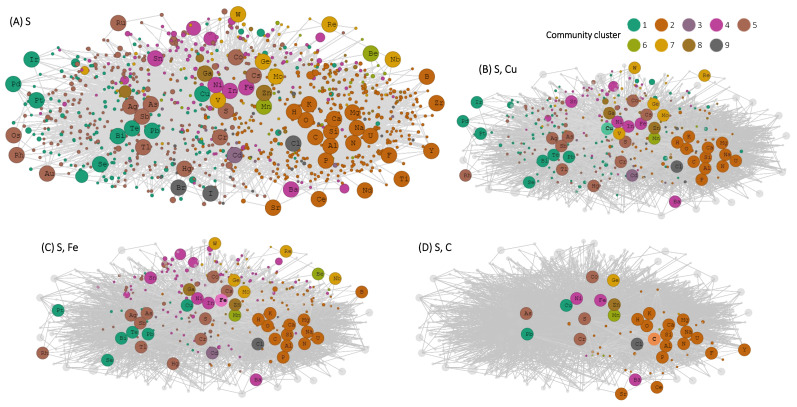
(**A**) Bipartite mineral chemistry network of all S-containing minerals. The network contains element nodes and mineral nodes. Element nodes are represented by circles with chemical element symbols, mineral nodes are represented by smaller unlabeled colored circles. Element and mineral nodes are colored by Louvain network community [36]. (**B**) Same network as 3A, but with only minerals that contain S and Cu highlighted. (**C**) Same network as 3A, but with only minerals that contain S and Fe highlighted. (**D**) Same network as 3A, but with only minerals that contain S and C highlighted.

**Figure 4:**
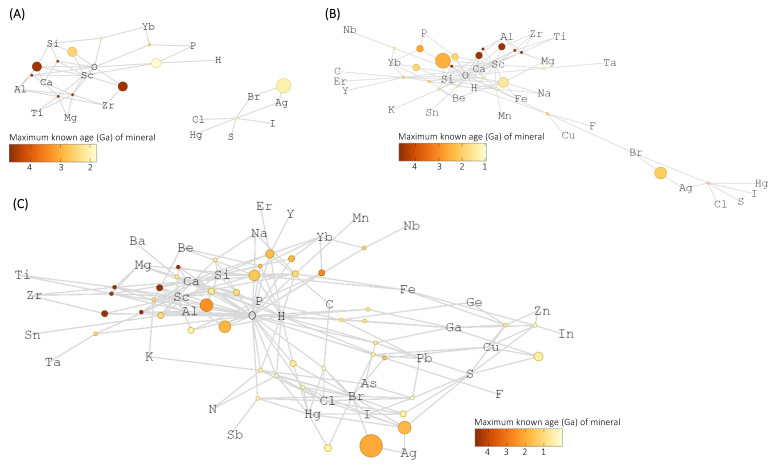
Combined bipartite mineral chemistry network of all Sc-containing minerals, Ga-containing minerals, Br-containing minerals, and Yb-containing minerals at (**A**) >1.7 billion years ago (Ga); (**B**) >0.6 Ga; (**C**) >0 Ga. Mineral nodes are colored by maximum known age.

Mineral chemistry networks provide greater insight into chemical associations of positive and negative outlier elements described above. Sulfur forms minerals with many different elements, but transition metals cluster separately from carbon (Figure 3). The expanding mineral network of negative outlier elements Sc, Ga, Br, and Yb prior to >1.7 Ga contains very few minerals (12 minerals), is not fully connected until 1.5 Ga, and does not exceed 40 minerals until present day (Figure 4). Sulfur only forms one mineral with Br at >1.7 Ga, and does not form minerals with any other focal elements (Sc, Ga, Yb) in the network until 0.541 Ga when the oldest known occurrences of gallite (CuGaS_2_) and gallobeudantite [PbGa_3_(AsO_4_)(SO_4_)(OH)_6_] appear in the geologic record. Sulfur remains a rare mineral-forming element with Sc, Ga, Br, and Yb despite the large number of total S-containing minerals. Minerals containing Sc and Br occur at the largest number of localities in the Sc, Ga, Br, Yb network. Abundant crustal elements O, Mg, Ca, and Si are the most common mineral-forming elements with Sc. Conversely, the mineral bromargyrite (AgBr) makes up the majority of known Br mineral localities.

**Figure 5:**
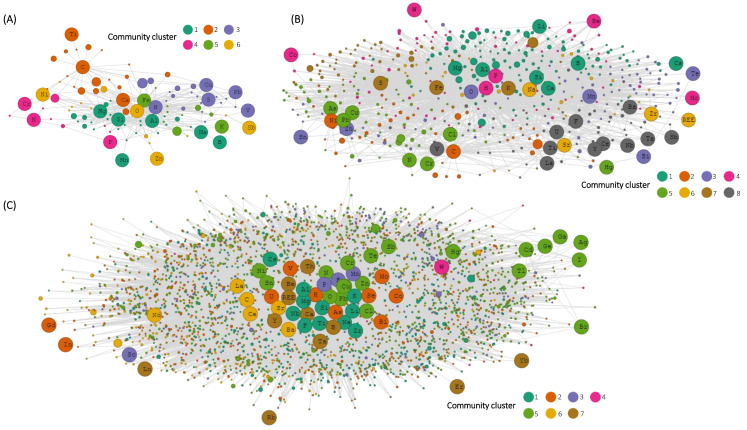
Combined bipartite mineral chemistry network of all H-containing minerals, C-containing minerals, and N-containing minerals at (**A**) >3.5 billion years ago (Ga); (**B**) >2.3 Ga; (**C**) >0 Ga. Element and mineral nodes are colored by Louvain network community [36].

The elements C, H, and N occur in separate network communities in the combined C, H, and N mineral chemistry network at >3.5 Ga (Figure 5A). At >2.3 Ga and >0 Ga, the number of H-containing minerals increased much more than C and N, but C and N overlap to a greater extent than they did at >3.5 Ga in terms of the elements they each separately form minerals with (Figure 5B). Carbon- and hydrogen-containing minerals are primarily carbonate hydroxides and carbonate hydrates. There are many more H-containing minerals (2708) than C-containing minerals (417) and N-containing minerals (85) in this analysis, and the combined H, C, N network is dominated by H-containing minerals at present day (Figure 5C). Overall, C, H, and N occur in a larger number of minerals and form minerals with larger numbers of other elements than the trace elements that are included in the recommended bulk crustal composition [2].

## 4. Discussion

Abundant crustal elements and trace crustal elements each played important roles in the geochemistry of early life [39,40,41]. Some of the oldest rocks on Earth located in the Isua supracrustal belt in Western Greenland [42,43,44,45] contain geochemical evidence of early life and primitive metabolism [46,47]. The strong correlation between #-mineral-elements and the number of mineral species that each element occurs in (Figure 1; [31,32,33]), including essential biological elements and trace element catalysts, indicates that essential biological elements, trace element catalysts, and non-biological elements follow the same trend of incorporation into crystal lattice mineral structures. As expected, elements that form minerals with a wider range of different elements form more mineral species and occur at more localities. The essential biological elements C, H, O, P, and S [48] form minerals with a larger number of other elements than most other non-biological elements. The propensity of these elements to spontaneously form ordered mineral crystal lattices, and also be involved in electron transfer reactions and acid base reactions involved in energy transfer, supports their essential biological roles. Iron is the most abundant metal in biology [49,50] and forms minerals with more elements and occurs in more mineral species than do any other transition metal, as well as the elements P, C, and N.

Despite following the correlation between mineral-forming elements and mineral species (Figure 1), N forms minerals with fewer elements and occurs in fewer mineral species, and at fewer mineral localities than do the other essential biological elements (Figure 1). The number of elements with which N forms minerals, the number of N-containing minerals, and the number of N-containing mineral localities all fall more in the range of many non-biological elements. Additionally, the number of elements that N forms minerals with is lower than any other element with an atomic number equal to or less than Ca (Figure 2B). The limited presence of N in minerals in the lithosphere is largely influenced by biogeochemical cycles, resulting in loss of N from the biosphere to the atmosphere, and contributing to the large inert atmospheric reservoir of volatile N_2_ and subsequent biological N limitation [51,52]. Additionally, in the oxidizing conditions of the upper mantle N exists primarily as N_2_ which is readily degassed, while in the deeper reducing mantle N primarily exists as NH_4_^+^ [53,54]. Ammonium is soluble in mantle minerals as a trace constituent [27,55], and N_3_^-^ can replace O_2_^−^ in certain silicate minerals [56], but N is not incorporated into mineral crystal structures to the same extent as other elements in the mantle due in part to its volatility [57,58].

The majority of mineral-forming elements show a positive association between the element’s crustal percent weight abundance and #-mineral-elements. This trend aligns with the fact that sufficient amounts of a mineral’s constituent elements need to be available for uniform crystal structures and natural kinds to form [37,59,60]. However, there are multiple outliers to this trend, including consequential biological elements (Figure 2A). Indeed, the low R^2^ value of the correlation between atomic number and #-mineral-elements is due in part to mineral element outliers with unique physical properties and mineralization trends such as N and Pb (Figure 2B). Sulfur, Cu, Pb, and As form minerals with more elements than expected based on their crustal abundance. Among these positive outliers S and Cu have positive biological utility and greater crustal abundance than Pb and As, which are biologically toxic. The same reactive properties of S, Cu, Pb, and As that result in these elements occurring in more mineral species than expected based on their crustal abundance likely also drive their biological utility or toxicity. 

Sulfur is an essential biological element [61,62,63], and protein active sites commonly include cysteine to provide sulfur-metal bonds and incorporate metal cofactor elements [64,65,66,67]. The S bonds therefore represent an interface between the organic amino acid protein sequence and the inorganic metal cofactor [68,69,70], which predominately includes elements with atomic numbers less than Zn (Figure 2B). Sulfur forms minerals with a wide range of elements, particularly transition metals and carbon (Figure 3A). However, transition metals such as Fe and Cu cluster separately from C in the S mineral chemistry network (Figure 3B–D). The S network community cluster 2, which contains C, also contains the biologically essential elements H, N, O and P, and biological trace elements potassium (K), calcium (Ca), magnesium (Mg), sodium (Na), silicon (Si), and aluminum (Al). There are 72 S-containing minerals that also contain both Fe and Cu, and 43 S-containing minerals that also contain C. The interactions of S with a wide range of different elements, including transition metals and carbon, in mineral chemical formulas coincide with the biological role of S to link the structural and redox properties of organic and inorganic elements [71,72]. Copper is also an important biological metal used as a cofactor in many protein catalytic sites, particularly proteins involved in energetically favorable aerobic metabolic pathways that evolved later than deeply rooted sulfur-based metabolisms [7,73,74].

The positive outlier elements Pb and As are mainly of consequence to biology due to their toxicity to a wide range of different types of organisms [75,76,77,78]. Indeed, the Great Oxidation Event (GOE) at 2.4 to 2.3 Ga [79] coincided with a sharp increase in arsenate (AsO_4_^3−^) and arsenic sulfides in marine shales, resulting in the emergence of a new selective pressure on the survival of marine microbial communities due to the widespread appearance of toxic, oxidized chemical species such as arsenate in seawater [80]. Furthermore, chondritic and non-chondritic meteorites in the early stages of solar system formation incorporate 41 different mineral-forming elements, including the earliest known appearances of mineral species that contain As, Pb, and the earliest known examples of galena (PbS), altaite (PbTe), and various arsenides [81,82,83]. Conversely, the elements Sc, Ga, Br, and Yb form minerals with fewer elements than expected based on their crustal abundance (Figure 2A). Scandium, gallium, bromine, and ytterbium are widely dispersed in the lithosphere, but rarely occur as a constituent element in the nominal formula of mineral species [84,85,86,87]. Specifically, Br is commonly a trace element in chlorine- (Cl) and iodine-containing minerals, rather than forming minerals containing Br in the nominal formula [88]. The combined Sc, Ga, Br, Yb network is not fully connected at 1.7 Ga, such that the single Br-containing mineral at this time does not contain any elements that also form minerals with Sc, Ga, or Yb (Figure 4A). Sulfur is a minor element in the combined Sc, Ga, Br, Yb network until 0.6 Ga (Figure 4). The combined Sc, Ga, Br, Yb network includes two distinct groups that contain either O or S, with limited overlap. Similarly, the elements Sc, Ga, Br, and Yb are also not important biological elements like the positive outlier elements S and Cu, and are not consequential toxic elements like the positive outlier elements Pb and As.

The essential biological elements C and N rarely form minerals together, such that ~95% of C-containing mineral species do not contain N and ~84% of N-containing mineral species do not contain C. Furthermore, there are no mineral species that contain both C and N that have a maximum known age >2.3 Ga (Figure 5). This highlights the importance of nitrogen and carbon fixation metabolic pathways to reduce gaseous nitrogen and oxidized carbon to biologically usable forms [51,89]. Indeed, the majority of minerals that contain both C and N derive from guano and other urine related sources over the Phanerozoic [38,90]. The combined C, H, N network at ≥2.3 Ga community cluster 5 that contains N only includes one biological metal element (Cu), but in the combined C, H, N network at >0 Ga the community cluster that contains N (community cluster 5) also includes the biological elements Cu, O, Zn, and Ni indicating greater chemical connection of biological elements with N (Figure 5). There are numerous different paragenetic processes (e.g., near-surface weathering/oxidation, subsurface hydrothermal deposition, and igneous lithologies) that lead to the formation of C-, H-, or N-containing minerals (described in great detail by [91]), and C and H form minerals in many more paragenetic modes than nitrogen. Hydrogen is the common electron source/carrier to reduce N and C (H comes from H_2_O for C fixation) in each of these processes [15,92]. Similarly, the vast majority of C-containing minerals (70.4%) and N-containing minerals (89.8%) also contain H, and all mineral species that contain both C and N also contain H. The geochemical trends of C, N, and H reflect biological redox relationships of these crucial elements.

## 5. Conclusions

Strong correlations between #-mineral-elements and the number of mineral species that an element occurs in, as well as between #-mineral-elements and the number of mineral localities of that element, highlight the relationships between geochemistry, the distribution of each element, and potential biological roles. Nitrogen forms minerals with fewer elements and occurs in fewer element species at fewer localities than the other essential biological elements (C, H, O, P, S), which reflects the unique biogeochemistry of N. The correlation between crustal percentage of each element and #-mineral-elements, and the biological importance or significance of positive outliers (S, Cu, Pb, As) vs. the inconsequential negative outliers (Sc, Ga, Br, Yb), reflects the link between geochemical reactivity and biological function/impact. The positive outlier element S is an important bridge element between organic (e.g., amino acids) and inorganic (metal cofactors) biological components. The element H is crucial in the reduction and biological fixation of N and C, and a common mineral forming element with N and C. Both abundant crustal percentage vs. #-mineral-elements insiders and less abundant outsiders have important biological functions as essential structural elements and catalytic cofactors. The unique properties that make specific chemical elements crucial to biology are also reflected in the chemistry and distribution of these elements in minerals and the geosphere. The trends observed in this study can help generate hypotheses for astrobiological studies on Earth and beyond investigating relationships between planetary geochemistry and prebiotic chemistry. Inherent reactivity and capacity for diverse ordered chemistry among biologically essential (C, H, N, O, P, S) and cofactor (Fe, Cu, Mn, Zn, Ni, Co, Mo) chemical elements reflected in the geosphere (mineral crystal lattices) and biosphere (amino acids and metal cofactors), under a wide range of environmental conditions that may be present on other worlds, support the possibility of the emergence of life elsewhere in the universe.

## Data Availability

All code and associated data needed to reproduce analyses are available from and documented in the repository https://github.com/spielmanlab/element_network_analysis; accessed on 3 February 2020. Crustal weight percent abundances calculated from Rudnick and Gao (2003) are available, along with associated code, in the R dragon package version >1.2.0 (https://github.com/sjspielman/dragon; accessed on 3 February 2020).

## Appendix A

**Table A1:** Mineral data for each chemical element included in this study: the number of elements that each element forms minerals with (#-Mineral-Elements); the number of minerals that each element occurs in the idealized chemical formula (Minerals); the number of localities that each element occurs at in mineral form; the Hard-Soft Acid-Base (HSAB) classification of each element; and the percent weight of each element in Earth’s crust (crust percent weight). Elements with “NA” Crust Percent Weight” did not have crust percent weight included in Rudnick and Gao (2003).

#-Mineral-					Crust Percent
Element	Element	Minerals	Localities	HSAB	Weight
Ag	28	172	3166	Soft acid	5.58 × 10^−8^
Al	52	881	10,322	Hard acid	0.083816762
As	59	634	4664	Hard acid	2.49 × 10^−6^
Au	12	31	5616	Soft acid	1.29 × 10^−9^
B	42	232	1295	Soft acid	1.10 × 10^−5^
Ba	40	218	2904	Hard acid	4.54 × 10^−4^
Be	36	121	1352	Hard acid	1.89 × 10^−6^
Bi	40	212	1821	Int. acid	1.79 × 10^−7^
Br	15	13	88	Soft base	8.76 × 10^−7^
C	50	417	9650	Soft base	NA
Ca	56	1152	11,362	Hard acid	0.045629216
Cd	20	25	253	Soft acid	7.97× 10^−8^
Ce	38	132	893	Hard acid	4.28 × 10^−5^
Cl	48	352	2219	Int. base	2.43 × 10^−4^
Co	24	67	1334	Int. acid	2.65 × 10^−5^
Cr	37	76	1115	Hard acid	1.34 × 10^−4^
Cs	28	23	82	Hard acid	1.99 × 10^−6^
Cu	57	667	9489	Int. acid	2.69 × 10^−5^
F	47	380	3775	Hard base	5.51 × 10^−4^
Fe	60	986	14,730	Int. acid	0.051949338
Ga	12	7	7	Hard acid	1.59 × 10^−5^
Ge	21	29	64	Hard acid	1.29 × 10^−6^
H	62	2708	13,356	Hard acid	NA
Hg	31	101	773	Soft acid	2.99 × 10^−8^
I	18	24	126	Soft base	6.97 × 10^−7^
In	14	10	46	Int. acid	5.18 × 10^−8^
Ir	11	13	71	Soft acid	3.69 × 10^−11^
K	49	440	6449	Hard acid	0.014965767
La	26	42	154	Hard acid	1.99 × 10^−5^
Li	31	115	728	Hard acid	1.59 × 10^−5^
Mg	47	583	6611	Hard acid	0.027989626
Mn	52	523	2492	Int. acid	7.71 × 10^−4^
Mo	32	64	2427	Int. acid	7.97 × 10^−7^
N	28	85	167	Int. base	NA
Na	48	923	4877	Hard acid	0.022740134
Nb	36	141	677	Hard acid	7.97 × 10^−6^
Nd	22	25	58	Hard acid	1.99 × 10^−5^
Ni	38	137	1590	Int. acid	5.88 × 10^−5^
O	66	3892	21,754	Hard base	0.462294236
Os	4	5	44	Soft acid	4.08 × 10^−11^
P	50	549	2931	Soft base	5.65 × 10^−4^
Pb	57	506	6728	Int. acid	1.10 × 10^−5^
Pd	15	68	184	Soft acid	1.49 × 10^−9^
Pt	14	26	217	Soft acid	1.49 × 10^−9^
RE	32	32	111	NA	NA
Rh	5	9	45	Soft acid	NA
Ru	5	6	60	Soft acid	5.98 × 10^−10^
S	59	1016	14,012	Int. base	4.02 × 10^−4^
Sb	43	235	2984	Hard acid	1.99 × 10^−7^
Sc	15	13	40	Hard acid	2.18 × 10^−5^
Se	30	118	370	Soft base	1.29 × 10^−7^
Si	54	1321	16,057	Hard acid	0.28214177
Sn	39	82	1384	Hard acid	1.69 × 10^−6^
Sr	35	118	651	Hard acid	3.19 × 10^−4^
Ta	29	54	347	Int. acid	6.97 × 10^−7^
Te	34	161	870	Soft base	NA
Th	28	33	497	Hard acid	5.58 × 10^−6^
Ti	42	312	3300	Hard acid	0.004298094
Tl	22	70	52	Soft acid	4.98 × 10^−7^
U	43	281	1694	Hard acid	1.29 × 10^−6^
V	42	206	882	Hard acid	1.37 × 10^−4^
W	34	37	1218	Int. acid	9.96 × 10^−7^
Y	40	115	650	Hard acid	1.89 × 10^−5^
Yb	10	5	8	Hard acid	1.89 × 10^−6^
Zn	44	255	6089	Int. acid	7.17 × 10^−5^
Zr	33	118	1482	Hard acid	1.31 × 10^−4^
